# Successful Extracorporeal Blood Purification Therapy using Double Haemoadsorption Device in Severe Endotoxin Septic Shock: A Case Report

**DOI:** 10.2478/jccm-2022-0028

**Published:** 2022-11-12

**Authors:** Stefano Ferraro, Stefania Bianzina, Sonila Mocka, Francesca Cappadona, Giovanni Battista Traverso, Fabio Massarino, Pasquale Esposito

**Affiliations:** 1 Unità di Nefrologia, San Paolo Hospital, Savona, Italy; 2 Neonatal and Pediatric Intensive Care Unit, Emergency Department, IRCCS Istituto Giannina Gaslini, Genova, Italy; 3 Unità di Nefrologia, Sant’Andrea Hospital, La Spezia, Italy; 4 Clinica Nefrologica, Dialisi, Trapianto, IRCCS Ospedale Policlinico San Martino, Genova, Italy; 5 Department of Internal Medicine, University of Genova, Genova, Italy

**Keywords:** acute kidney injury, continuous renal replacement therapy, sepsis, haemoadsorption

## Abstract

**Introduction:**

In patients admitted to the Intensive Care Unit (ICU), sepsis can lead to acute kidney injury (AKI), which may require the initiation of continuous renal replacement therapy (CRRT) in 15-20% of cases. There is no consensus about the best extracorporeal treatment to choose in septic patients with AKI.

**Case presentation:**

We describe the case of a 70-year-old woman admitted to the ICU with a severe endotoxin septic shock due to Neisseria meningitidis serogroup C. Despite prompt medical intervention, including fluid resuscitation, high dose vasopressor, inotrope support, and broad-spectrum antimicrobial treatment, in a few hours patient’s haemodynamic worsened and she developed multi-organ failure, including severe AKI, requiring CRRT. So, continuous veno-venous haemodiafiltration was started, using an oXiris® haemodiafilter set, in series with an adsorber device (CytoSorb®). After 48 hours of this combined extracorporeal treatment, haemodynamic parameters improved, allowing a significant reduction of the vasoactive therapy, with a concomitant decrease in endotoxin and inflammatory markers serum levels. In the following days patient’s conditions still improved and renal function recovered.

**Conclusions:**

Timely extracorporeal blood purification therapy, using a double haemoadsorption device, may be effective in the management of severe septic shock.

## Introduction

Neisseria meningitidis is a gram-negative diplococcus, that constitutes the leading cause of meningitis, with an overall incidence in Europe and the USA of 1-3 per 100,000 population. Clinical disease is often associated with rapid onset of fever, headache, and a non-blanching haemorrhagic rash, which is pathognomonic for meningococcal disease. The mortality rate remains 10 to 15% for all cases while rising to 40% in patients with severe sepsis [1].

One of the main mechanisms of sepsis-related systemic damage is the dysregulation of the host immune response against the infection. It is mediated by the presence of bacterial cell wall components, known as pathogen-associated molecular patterns (PAMPs), such as endotoxin or lipopolysaccharide, and the elements of the innate immune response, known as damage-associated molecular patterns (DAMPs). These patterns represent triggers for an exaggerated immune response, leading to pro/anti-inflammatory cytokines release, which determines the so-called “cytokine storm” [2]. These findings support the hypothesis that, beyond antibiotic therapy and infection source control, a possible additive strategy in sepsis treatment could be the restoring of a balanced immune response, by

removing inflammatory mediators from the bloodstream.

Sepsis is associated with a high mortality rate and multi-organ failure (MOF), including acute kidney injury (AKI), which requires continuous renal replacement therapy (CRRT) in about 20% of patients admitted to the Intensive Care Unit (ICU) [3]. However, there is no consensus about the best extracorporeal treatment to choose in septic patients with AKI. Indeed, it has been suggested that CRRT could not only be useful to treat renal failure itself but also to modulate sepsis-related immune dysregulation, using specific blood purification techniques, like haemoperfusion or other extracorporeal purification modalities. However, available data from clinical studies are contrasting, and a clear strategy is not yet defined [4].

Here, we present the case of a severe endotoxin septic shock due to Neisseria meningitidis serogroup C, which was treated with continuous veno-venous hae-modiafiltration (CVVHDF), using a combination of two different haemoadsorption devices.

## Case Report

A 70-year-old woman, without any prior confirmed medical history, was admitted to the Emergency Department with fever (39.6 °C), altered neurological status (Glasgow Coma Scale 10) without meningeal involvement, and non-blanching haemorrhagic rash. Laboratory examinations showed increased inflammatory markers (white blood cell count -WBC -15.46 x 103/μL, C-reactive protein - CRP - 122 mg/L), lactic acidosis (pH 7.26, lactate 6.2 mmol/L), elevated serum creatinine (SCr 1.5 mg/dL), hyponatremia (Na^+^ 133 mmol/L), hyperkalemia (K^+^ 5.7 mmol/L), thrombocytopenia and altered coagulative parameters. Total body CT scan showed inflammatory pulmonary lesions and disseminated lymphadenomegaly. Suspecting invasive meningococcal disease, empirical broad-spectrum antimicrobial therapy with ceftriaxone and ampicillin was introduced.

The patient rapidly deteriorated and developed a severe septic shock with MOF. Then, she was admitted to the ICU, with a Sequential Organ Failure Assessment (SOFA) score of 15, where she was immediately intubated, and mechanical ventilation was started. Due to haemodynamic instability (mean arterial pressure 45 mmHg), following the Surviving Sepsis Campaign Guidelines [5], fluid resuscitation with crystalloids at the dose of 30 ml/ kg was started, combined with vasopressor and inotropic support, rapidly titrated to high doses (norepinephrine 1 μg/kg/min, dobutamine 10 μg/kg/min and epinephrine 1 μg/kg/min). Moreover, the patient became anuric and sCr raised to 2.2 mg/ dL, accompanied by a prominent elevation of inflammatory markers (WBC 29.5 x 103/μL, CRP 146 mg/ L, procalcitonin - PCT - 559 ng/ml), as shown in Table 1. Endotoxin shock was confirmed by endotoxin activity assay (EAA) of 0.71. Neisseria meningitidis was detected by Real-Time - Polymerase Chain Reaction on blood samples and blood cultures subsequently confirmed the presence of Neisseria meningitidis serogroup C. Consequently, after infectious disease consultation, it was decided to continue antibiotic therapy with ceftriaxone.

**Table 1:** Time course of haemodynamic parameters and laboratory examinations during double haemoadsorptive CRRT treatment.

	Day 1					Day 2			Day 3	
Parameters	H.5.00 E.R.add.	H 7.00 ICU add.	H 8.00	H 12.00 Start double adsorption	H 23.00	H 8.00	H 12.00 End of Cytosorb 1-st cycle	H 19.00	H 8.00	H 12.00 End of Cytosorb 2-nd cycle
MAP (mmHg)	72	45	66	58	47	62	58	82	80	90
HR (bpm)	110	117	100	100	117	115	120	113	84	80
H/UO (ml/h)	300	100	-	100	0	0	0	0	0	0
DB (μg/kg/min)	0	0	6	10	10	10	10	2	0	0
NE (μg/kg/min)	0	0.5	0.5	0.7	1	1	1	1	1	1
E (μg/kg/min)	0	0	0.6	1	1	1	1	0.6	0.4	0.4
UFR (ml/h)	-	-	-	0	0	0	50	50	50	150
WBC, (103/μL)	15.460	29.500	-	29.400	-	25.550	24.880	-	19.440	-
Platelets, (103/μL)	123.000	53.000	-	53.500	-	17.000	22.000	-	43.000	-
CRP (mg/L)	122	146	-	145	-	354	301	-	258	-
PCT (ng/ml)	-	559	-	-	-	493	460	-	396	-
EAA, (Unit)		0.71								0.45
SCr, (mg/dl)	1.5	2.2	-	2.2	-	1.6	1.3	-	1.2	-

Abbreviations: CRRT, continuous renal replacement therapy; MAP, mean arterial pressure; HR, heart rate (beats per minute); H/UO, hour/urinary output; DB, dobutamine; NE, norepinephrine; E epinephrine; UFR, ultrafiltration rate; WBC, white blood cells; CRP, C-reactive protein; PCT, procalcitonin; EAA, endotoxin activity assay; SCr, serum creatinine; ER add, Emergency Room admission; ICU add, Intensive Care Unit admission.

In addition, after a multidisciplinary evaluation, because of the presence of endotoxin septic shock and severe AKI, CVVHDF treatment was started, using a modified AN69ST membrane (oXiris®, Baxter, Meyzieu, France), combined with CytoSorb® (CytoSorbents Corporation, Monmouth Junction, NJ, USA), an unselective porous particle adsorbing cartridge, for two consecutive cycles, using each cartridge for 24 hours. These two devices were incorporated in series on a CRRT platform (Prismaflex®, Baxter, USA) with post-dilution reinfusion. CVVHDF parameters were initially set to reach a prescribed dose of 40 ml/kg/h, with a convective dose of 25 ml/kg/h, without ultra-filtration. CRRT was started using systemic heparin anticoagulation. Haemodynamic parameters began to improve after the first 24 hours of treatment, allowing a progressive reduction of the inotropic support and the beginning of ultrafiltration (UF), at an initial rate of -50 ml/h. Patient’s haemodynamic further improved after the second cycle of continuous haemoadsorption treatment, with the possibility of discontinuing dobutamine therapy, halving the epinephrine dose, and concurrently increasing the UF rate to -150 ml/h. Moreover, this treatment resulted in a significant decrease in serum endotoxin levels (EAA 0.45) and inflammatory markers (Table 1). After 48 hours, CytoSorb® treatment was stopped, while CVVHDF was continued only with oXiris® membrane.

During the following days, the haemodynamic conditions further improved, allowing progressive weaning from vasoactive therapy. The patient was successfully extubated on day 15. From day 20, renal function started to improve, and diuresis increased (from anuria to 0.5 ml/kg/h), then CRRT was gradually discontinued and definitively stopped on day 31 (for a total of 9 CRRT sessions).

After a rehabilitation period, on day 67 the patient was discharged from the hospital.

At that time, the patient provided written consent to data collection and publication

## Discussion

Endotoxin septic shock is an extremely severe clinical condition, which can induce MOF, including AKI, associated with high morbidity and mortality. Thus, a prompt diagnosis and timely treatment are crucial in reducing mortality and improving patient outcomes. However, while antimicrobial treatment and infection source control remain the mainstay of therapy in sepsis, some patients develop severe MOF, that may require supportive extracorporeal therapies. Notably, the concept that extracorporeal techniques might be used not only to support organ failure but even as immunomodulatory therapy has emerged in recent years [4]. In our case, we decided to start CVVHDF treatment using the AN69ST oXiris® membrane, due to its peculiar properties, compared to other alternative treatments, such as high cut-off haemodialysis or high-volume haemofiltration. Indeed, AN69ST oXiris® has elevated adsorptive cytokine capacity due to the presence of methallylsulfonate, which provides a negative charge to the membrane [6]. Moreover, it is coated with polyethyleneimine, a cationic molecule that is useful to remove the endotoxin, and with pre-immobilized heparin, which ensures an anti-thrombogenic capacity. These properties were confirmed by both experimental and clinical studies [7].

From a clinical perspective, Shum et al. reported a reduction of SOFA score and vasopressor dose when oXiris® was used [8]. Similarly, Turani et al. found that oXiris was safe and improved the cardiorenal function and the general clinical conditions in patients with septic shock [9].

In our patient, because of the critical septic shock and the high vasopressor and inotropic requirement, we decided to use CytoSorb® treatment in addition to oXiris®, to improve the host immune response and haemodynamic status, by intensively removing inflammatory mediators. CytoSorb® is a polystyrene-based haemoadsorption device, able to reduce the serum levels of molecules ranging from 5 to 60 kDa [10].

It has been observed that extracorporeal blood purification with CytoSorb® effectively removes inflammatory cytokines and DAMPs in patients with sepsis and, recently, this device has been also studied as a supportive treatment for patients affected by Coronavirus disease 2019 (COVID-19) [11].

However, it should be also recognized that, despite its capacity to remove potentially harmful molecules, the actual impact of CytoSorb® on clinical outcomes remains in debate [12].

Nevertheless, in our patient, the combination of these two haemoadsorption techniques was associated with an initial improvement in the hemodynamic profile after the first 24 hours of treatment, followed by a further and more significant benefit after the second cycle. These improvements allowed a reduction in vasoactive support, together with a decrease in serum endotoxin levels and inflammatory markers.

## Conclusions

According to the limited literature data and our clinical experience, we strongly believe that extracorporeal renal replacement therapy, associated with haemoadsorption blood purification techniques, may be useful in managing severe septic shock associated with MOF. While waiting for specifically designed clinical studies, we suggest that the personalization of therapy, the choice of appropriate timelines, and the adoption of hybrid methods can help in reducing the mortality rate of endotoxic septic shock.
